# Systematic review of quality of life measures in patients with endometriosis

**DOI:** 10.1371/journal.pone.0208464

**Published:** 2019-01-10

**Authors:** Nicolas Bourdel, Pauline Chauvet, Valentina Billone, Giannis Douridas, Arnaud Fauconnier, Laurent Gerbaud, Michel Canis

**Affiliations:** 1 Department of Gynaecological Surgery, Clermont-Ferrand University Hospital, Clermont-Ferrand, France; 2 Faculty of Medecine, ISIT-University of Auvergne, Clermont-Ferrand, France; 3 Department of Mother and Child, University Hospital P. Giaccone, Palermo, Italy; 4 Department of Gynecology and Obstetrics, Centre Hospitalier Intercommunal de Poissy-Saint-Germain-en-Laye, Poissy, EA 7285 Research Unit ‘Risk and Safety in Clinical Medicine for Women and Perinatal Health’, Versailles-Saint-Quentin University (UVSQ), Montigny-le-Bretonneux, France; 5 Dept of Public Health, PEPRADE, Université Clermont Auvergne, CHU Clermont-Ferrand, France, CNRS, SIGMA Clermont, Institut Pascal, Clermont-Ferrand, France; Iranian Institute for Health Sciences Research, ISLAMIC REPUBLIC OF IRAN

## Abstract

**Objectives:**

Endometriosis and quality of life has been the subject of much research, however, there is little consensus on how best to evaluate quality of life in endometriosis, resulting in many and diverse scales being used. In our study, we aim to identify quality of life scales used in endometriosis, to review their strengths and weaknesses and to establish what would define an ideal scale in the evaluation of endometriosis-related quality of life.

**Materials and methods:**

A search of the MEDLINE and EMBASE databases was carried out for publications in English and French for the period from 1980 to February 2017, using the words ‘endometriosis’ and ‘quality of life’. Publications were selected if they reported on quality of life in patients with endometriosis and specified use of a quality of life scale. A quantitative and a qualitative analysis of each scale was performed in order to establish the strengths and weaknesses for each scale (systematic registration number: PROSPERO 2014: CRD42014014210).

**Results:**

A total of 1538 articles publications were initially identified. After exclusion of duplicates and application of inclusion criteria, 201 studies were selected for analysis. The SF-36, a generic HRQoL measure, was found to be the most frequently used scale, followed by the EHP-30, a measure specific to endometriosis. Both perform well, when compared with other scales, with scale weaknesses offset by strengths. EHP-5 and EQ-5D also showed to be of good quality. All four were the only scales to report on MCID studied in endometriosis patients.

**Conclusion:**

For clinical practice, routine evaluation of HRQOL in women with endometriosis is essential both for health-care providers and patients. Both SF-36 and EHP-30 perform better overall with regard to their strengths and weaknesses when compared to other scales.

## Introduction

### Endometriosis and quality of life

Endometriosis is a benign chronic disease affecting young women. One of the main symptoms is pain and endometriosis has a major impact on fertility. Three types of pain are generally associated with endometriosis (dysmenorrhea, deep dyspareunia and non-menstrual chronic pelvic pain [1]) though other symptoms may be present such as dyschezia, lower back pain and urinary symptoms. In addition, women with endometriosis experience a range of non-clinical symptoms including depression, feelings of isolation, fatigue and lack of energy. Endometriosis is reported to have an adverse impact on physical, mental, and social wellbeing [2] and a negative effect on health-related quality of life (HRQoL) [3].

Health-related quality of life is a multidimensional concept encompassing physical, psychological and social aspects associated with a particular disease or its treatment [4]. Only a few studies have specifically analyzed quality of life in patients with endometriosis [5] [6] [7] [8] with clinicians facing a common dilemma as to how to adequately evaluate HRQoL in patients with endometriosis.

HRQoL in women with endometriosis is a growing concern, increasingly voiced by health professionals and patients alike [9]. However, with little consensus on how best to evaluate quality of life in endometriosis a lots of scales have been used. This review aims firstly to identify the range of HRQoL instruments described in the literature and used in clinical endometriosis studies, secondly to analyze the main strengths and weaknesses of each instrument and finally, to determine what defines an ideal scale for clinicians and researchers in the evaluation of endometriosis-related quality of life.

## Methods

### Literature search

A computerized search of PubMed and EMBASE ressources was performed to identify all registered articles about endometriosis and QoL published between January 1980 and February 2017, using the following terms: “endometriosis and quality of life”, and “endometriosis and scale” (e.g. “endometriosis” and “EHP-30”, and “endometriosis” and “EHP30”). We included clinical trials, comparative studies, controlled and randomized controlled trials and multicenter studies. We excluded abstracts, commentaries and editorial publications. Publications were selected if they investigated endometriosis and quality of life, if a quality of life scale was used and specified and if they were reported in English or French. 201 articles were selected on the basis of inclusion criteria and cross-references checked. Two researchers collected the data independently, with data verified by a third researcher in cases of data disparity. The objective was to identify and provide detailed analysis of the varied assessment instruments used in evaluation of quality of life, including identification of their strengths and weaknessess. Analysis was first performed separately for each scale followed by a comparative analysis between the scales (systematic registration number: PROSPERO 2014:CRD42014014210).

### Description and comparison of scales

Guidelines for evaluation of the validity of a HRQoL questionnaire highlight **eight quality criteria** [10] [11]: test of data quality including **floor and ceiling effects** (the number of respondents obtaining the lowest or highest possible score),**content validity** (the extent to which the domain of interest is comprehensively sampled by questionnaire items), **internal consistency** (the extent to which items in a (sub)scale are intercorrelated, thus measure the same construct), **criterion validity** (the extent to which questionnaire scores relate to a gold standard), **construct validity** (the extent to which scores relate to other measures and are consistent with theoretically derived hypotheses relating to the concepts measured), **reproducibility (**the degree to which repeated measurements in stable persons provide similar answers)**,** r**esponsiveness** (the ability to detect changes over time), and **interpretability** (the degree to which qualitative meaning can be assigned to quantitative scores).

Other guidelines referred to by the authors included those published by the FDA (U.S. Department of Health and Human Services FDA, 2009) which emphasise the use of patient-reported outcomes (PRO) and instruments for evaluation of the safety and effectiveness of medical products, such as treatment for endometriosis or endometriosis-related Quality of Life.

On the basis of the aforementioned criteria, and taking into consideration the specificity of endometriosis, the following six areas were chosen for assessment of quality of life scales:

1. scale description and application
2. validity, responsiveness, reproducibility and reliability
3. disease specificity;
4. respondent and investigator burden and feasibility
5. validation in foreign languages
6. responder concept and MCID (Minimal Clinically Important Difference) after treatment

Appraisal of the methodological quality of the studies was done using the Canadian Task Force, a measurement tool to assess the methodological quality of studies. We evaluated the studies for all the scales, except the Self developed studies.

## Results

**Fig 1:**
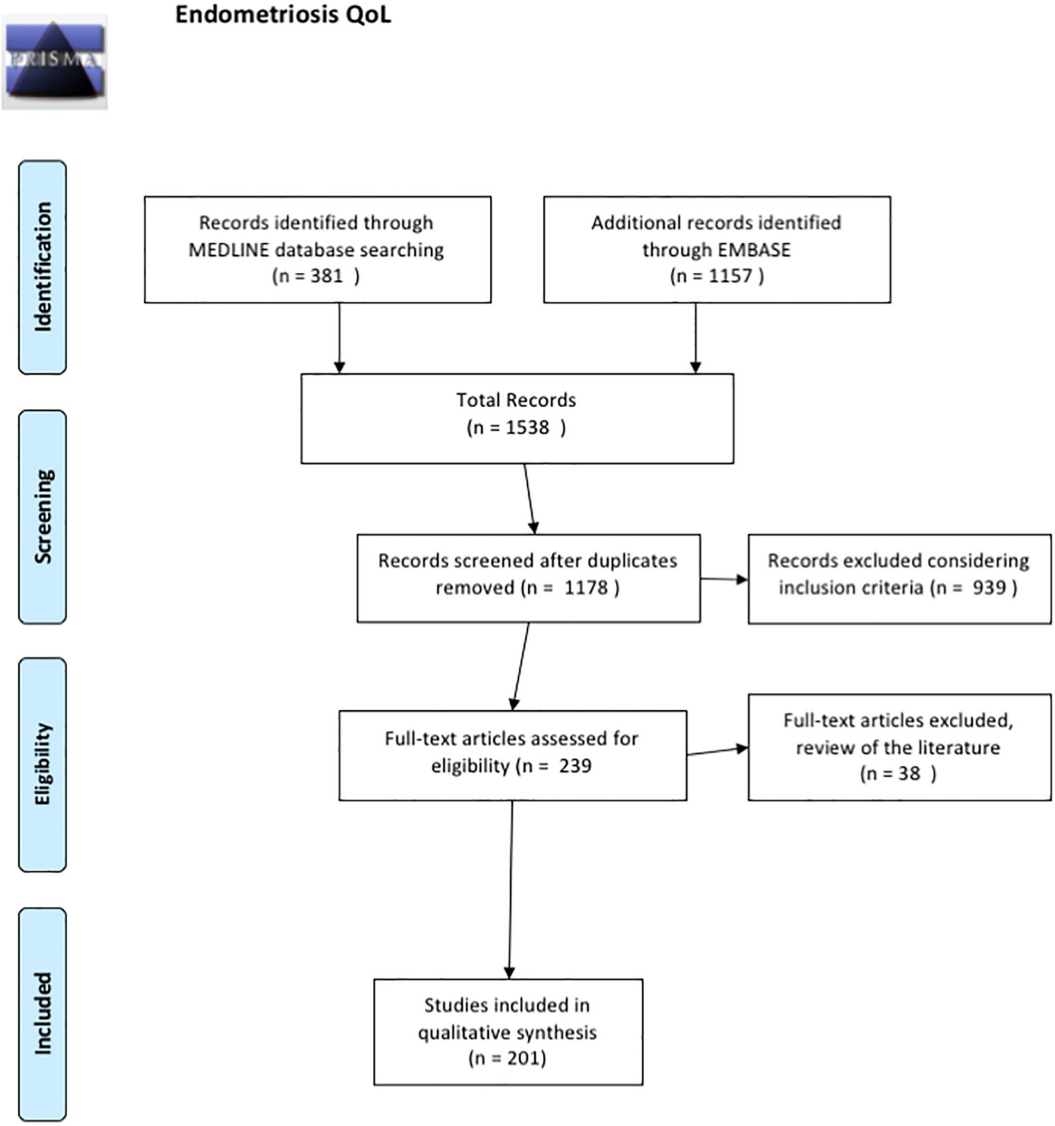
Flow diagram.

An initial search using the key words ‘endometriosis’, and ‘quality of life’, followed by a search using these key words preceded by the name of a scale, allowed identification of 1538 articles (Fig 1) from which 360 duplicates were excluded. Screening of abstracts for the remaining 1178 articles revealed that 939 did not address issues relating to endometriosis and quality of life or conform to inclusion criteria, with inadequate descriptions of quality of life scales used.

Following examination of the full-length text, 38 articles were excluded as they failed to specify the HRQoL scale used. **201 articles were retained for analysis**.

### HRQoL scales and quantitative analysis

From the 201 selected publications, 24 scales were identified as described below. Relevant references concerning each scale are presented in Tables 1 and 2.

**Table 1:** Studies found in a systematic review of endometriosis quality of life assessment and the quality of life scale used: The generic instruments.

Scale	Study references
**SF-36 (The Short-Form-36 Health Survey) n = 72**	Agarwal et al., 2015 [97], Angioni et al., 2015 [98], Angioni et al., 2015 [99], Augusto et al., 2016 [100], Bassi et al, 2011 [101], Berner et al. 2015 [102], Bodner et al., 1997 [43], Caruso et al., 2015 [103], Caruso et al., 2016 [104], Caruso et al., 2015 [105], Centini et al., 2013 [106], Chene et al, 2008 [107], Chene et al, 2012 [108], Chuamoor et al, 2012 [109], Darai et al, 2015 [75], Darai et al, 2009 [110], Darai et al, 2010 [111], De Graaf et al., 2013 [112], De Graaf et al, 2015 [113], Deguara et al, 2013 [114], Di Donato et al, 2013 [115], Dubernard et al, 2008 [6], Dubernard et al, 2006 [116], Farfaras et al, 2014 [117], Friedl et al, 2015 [118], Friggi Sebe Petrelluzzi et al., 2012 [119], Grandi et al, 2015 [120], Hefler et al, 2005 [121], Hong et al, 2014 [122], Jia et al, 2013 [40], Jones et al, 2004 [4], Jones et al, 2001 [36], Laas et al, 2015 [123], Laursen et al, 2005 [124], Leplege et al, 1998 [14], Lövkvist et al, 2016 [125], Mabrouk et al, 2012 [126], Mabrouk et al, 2011 [127], Mabrouk et al, 2011 [128], Maiorana et al, 2012 [129], Marques et al, 2004 [130], Piketty et al, 2007 [131], Melis et al, 2014 [132], Miller et al, 2000 [133], Montanari et al, 2013 [134], Morotti et al, 2014 [135], Nnoaham et al, 2011 [85], Nogueira-Silva et al, 2015 [69], Nojomi et al, 2011 [39], Nunes et al, 2014 [136], Petrelluzzi et al, 2008 [137], Pontis et al, 2016 [138], Remorgida et al, 2007 [139], Ribeiro et al, 2014 [140], Roman et al, 2015 [141], Roman et al, 2012 [142], Rubi-Klein et al, 2010 [143], Gallagher et al, 2016 [144], Sesti et al, 2007 [145], Siedentopf et al, 2008 [146], Silveira da Cunha Araùjo et al, 2014 [147], Strowitzki et al, 2010 [148], Strowitzki et al, 2010 [149], Strowitzki et al, 2012 [150], Stull et al, 2014 [60], Tanmahasamut et al, 2012 [151], Teixeira et al, 2017 [152], Touboul et al, 2015 [74], Tu et al, 2014 [153], Vercellini et al, 2002 [154], Zhao et al, 2012 [155], Zupi et al, 2004 [156].
**SF-12 (The Short-Form-12 Health Survey) n = 16**	Abbott et al, 2004 [66], Abbott et al, 2003 [157], Carey et al, 2014 [158], De Graaf et al, 2016 [159], Deguara et al, 2013 [114], Di Francesco et al, 2014 [160], Facchin et al, 2015 [161], Fourquet et al, 2011 [162], Garry et al, 2000 [3], Luisi et al, 2015 [163], Lyons et al, 2006 [164], Meissner et al, 2016 [165], Moawad et al, 2011 [166], Soto et al, 2017 [167], Vercellini et al, 2016 [168], Zupi et al, 2015 [169]
**EQ-5D (The European Quality of Life–5 Dimensions questionnaire) n = 24**	Abbott et al, 2004 [66], Bailly et al, 2013 [170], Bluett et al, 2016 [184], Daniels et al, 2009 [171], English et al, 2007 [172], English et al, 2014 [173], Ford et al, 2004 [174], Fritzer et al, 2014 [175], Gao et al, 2006 [78], Garry et al, 2000 [3], Kent et al, 2016 [185], Klein et al, 2014 [176], Li et al, 2014 [177], Lyons et al, 2006 [164], Meuleman et al, 2011 [178], Michalak et al, 2016 [186], Radosa et al, 2016 [187], Radosa et al, 2014 [179], Roman et al, 2010 [180], Roman et al, 2010 [181], Shakir et al, 2015 [188], Simoens et al, 2012 [8], Simoens et al, 2011 [182], The Luna trial collaboration, 2003 [183].
**NHP (The Nottingham Health Profile) n = 3**	Bergqvist et al, 2001 [18], Burry et al, 1992 [19], Clarke et al, 1995 [20]
**WHOQOL-BREF (**The World Health Organization Quality of Life bref) **n = 11**	Cao et al, 2015 [189], Giuliani et al, 2016 [88], Kiykac Altinbas et al, 2015 [190], Lee et al, 2016 [191], Leonardo-Pinto et al, 2017 [192], Lin et al, 2014 [193], Sepulcri et al, 2009 [194], Souza et al, 2011 [195], Tripoli et al, 2011 [89], Yela et al, 2015 [196], Zhao et al, 2013 [197]
**Ferrans & Powers’ Quality of Life Index (QLI) n = 2**	Rannestad et al, 2001 [34], Laganà et al, 2015 [198]
**Duke Health Profile** [24] n = 3	Stratton et al, 2008, 2015 [26,27], Karp et al, 2011 [25]
15D n = 3	Taipale et al, 2009 [30], SetäLä et al, 2012 [28], Kössi et al, 2013 [29]

**Table 2:** Studies found in a systematic review of endometriosis quality of life assessment and the quality of life scale used: The specific scales and self-developed measures.

Scale	Study references
**EHP-30 (**The 30-item Endometriosis Health Profile) **n = 43**	Ahn et al, 2009 [199], Al-Azemi et al, 2009 [200], Benbara et, 2008 [201], Chauvet et al, 2017 [38], Cheong et al, 2014 [202], Daraï et al, 2009 [110], Flower et al, 2011 [203], Friedl et al, 2015 [118], Gallicchio et al, 2015 [204], Gao et al, 2006 [78], Gonçalves et al, 2017 [205], Hansen et al, 2013 [82], Hansen et al, 2014 [83], Jenkinson et al, 2008 [206], Jia et al, 2013 [40], Jones et al, 2001 [36], Jones et al, 2004 [4], Jones et al, 2004 [207], Jones et al, 2006 [7], Kent et al, 2016 [185], Khong et al, 2010 [79], Maiorana et al, 2012 [129], Meuleman et al, 2011 [178], Meuleman et al, 2011 [208], Meuleman et al, 2009 [5], Meuleman et al, 2014 [209], Middleton et al, 2017 [210], Mira et al, 2015 [211] Nogueira-Silva et al, 2015 [69], Nojomi et al, 2011 [39], Protopatas et al, 2014 [212], Rostami et al, 2015 [213], Selvi-Dogan et al, 2016 [214], Shakir et al, 2015 [188], Soliman et al, 2017 [215], Soto et al, 2017 [167], Tan et al, 2013 [216], Van de Burgt et al, 2011 [41], Van de Burgt et al, 2013 [63], Van der Houwen et al, 2014 [217], Vercellini et al, 2013 [218], Wayne et al, 2008 [219], Wickström et al, 2013 [220],
**EHP-5 (**The 5-item Endometriosis Health Profile) **n = 12**	Acs et al, 2015 [221], Bailly et al, 2013 [170], Boileau et al, 2012 [222], Carr et al, 2014 [223], Fauconnier et al, 2017 [62], Fourquet et al, 2011 [162], Goshtasebi et al, 2011 [70], Goyal et al, 2016 [224], Jones et al, 2004 [207], Minas et al, 2014 [225], Renouvel et al, 2009 [226], Selcuk et al, 2015 [71].
Self developed questionnaires (n = 1 for each scale)	Oehmke et al. 2009 [46] Trehan and Sanaullah 2009 [47] Kumar et al. 2011 [49] Learman et al. 2011 [52] Ceccaroni et al. 2012 [54] Issa et al. 2012 [55] Fritzer et al. 2012 [57] Chapron et al, 2015 [58] Regidor et al. 1997 [45] Mathias et al. 1996 [44] Bodner et al. 1997 [43] Colwell et al. 1998 [42]

#### Generic scales

**SF-36: The Short-Form-36 Health Survey (n = 72).** The SF-36 (or MOS (Medical Outcome Study) Short Form-36) survey was the most commonly used scale, appearing in a total of 252 publications, of which 72 studies were included in our analysis. The SF-36 derived from the Rand Health Insurance Experiment [12], a survey made by the RAND Corporation during the 1970’s, in order to provide a HRQoL measure filling the usual endpoints used to assess medical outcomes. It was developed in the 1980’s and published by Ware J et al in 1992 [13]. The initial MOS surveys covered 40 physical and mental health concepts, from which shortened versions were developed. The SF-36 is a generic health status measurement instrument and as such can be used to assess health-related quality of life, independent of the disease affecting the population under study. It is comprised of 36 items, one of which concerns health transition and the remaining 35 items correspond to eight health scales: physical functioning, role limitations relating to physical health, bodily pain, general health perceptions, vitality, social functioning, role limitations relating to mental health, and mental health.

The SF-36 is easy to use and score, with subjects obtaining a score between 0 and 100 for each health scale, where higher values indicate better HRQoL. It can be self-administered or administered by personal interview or by telephone, taking on average 5-10minutes to complete. Subject responses are presented as a profile of scores corresponding to each scale. The survey has been widely tested internationally and translated into many languages by the International Quality of Life Assessment (IQOLA) project [14]. The SF-36 has been validated for endometriosis and is considered a valid and responsive measure for endometriosis and its treatment [15]. A mapping of the 6 scales (SF6D) can be used to calculate Quality Adjusted Life Years (QuALY) in technology assessment.

**SF-12 The Short-Form-12 Health Survey (n = 16). (***John E*. *Ware*, *1994 Jr*. *revised 1998)*. A total of 199 publications were identified as using the SF-12, of which 16 studies were included in our analysis**.** The 12-Item Short Form Health Survey (SF-12) is a short form of the SF-36, also developed for the MOS. It was designed to be broad ranging, less sensitive to patients’ conditions, sufficiently brief for use in large-scale surveys, while still providing physical and mental scores as for the SF-36. It is generally used in surveys and outcome studies where time constraints prevent use of the SF-36. It can be also used to compute SF6D.

**EQ-5D: The European Quality of Life–5 Dimensions questionnaire (n = 24).** The European Quality of Life–5 Dimensions questionnaire **(EQ-5D)** was identified in a total of 55 publications**,** of which 24 studies were included in the analysis. It is a generic HRQoL instrument, developed by the EuroQol Research Foundation, and as its name suggests, measures quality of life using five dimensions (mobility, self-care, usual activities, pain/discomfort, anxiety/depression). Responses correspond to one of three levels of severity (no problems/some or moderate problems/extreme problems). The EQ-5D is a descriptive system. Assessment of descriptions leads to health status expressed initially as a 5-digit number which can be weighted according to preferences and converted into a single weighted index score. Applicable to a wide range of health conditions and treatments, the EQ-5D health questionnaire provides a simple descriptive profile and a single aggregated index value for health status. But, it is worthy to note that it mixes questions related to disability (questions 1 to 3) and two questions actually realted to quality of life. The EQ5D is mainly used to compute QuALY in technology assessment, as recommended by the British NICE or the French HAS. It’s been translated in many languages, and it is taking only a few minutes to complete. It can be used as self questionnaire, or filled in with the help of a surveyor.

**The NHP: The Nottingham Health Profile (n = 3)**. The Nottingham Health Profile (NHP) was designed to give a brief indication of perceived physical, social and emotional health problems [16]. Originally intended for use in primary medical care settings, it has also been used to assess need for care in health surveys and as an outcome measure in clinical trials [17].

The original version, known as the Nottingham Health Index, contained 33 items. The revised version, called NHP, is composed of two parts. Part I contains 38 items divided into six sections: physical abilities (8 items), pain (8 items), sleep (5), social isolation (5), emotional reactions (9) and energy level (3 items). Part II provides a brief indicator of handicap and contains seven items relating to the effect of health problems on employment, jobs around the house, personal relationships, social and sex life, hobbies and holidays. Part II is optional and more rarely used, due to some items (e.g. work, sex life) not always being applicable. All questions have only yes/no answer options and each section score (maximum 100) is weighted. Higher scores indicate a greater number and severity of problems. The NHP is self-administered and takes five to ten minutes to complete. In the present analysis, 3 studies exploiting the NHP were included [18–20] with the latter only using a modified version of Part II.

**WHOQOL-BREF (n = 11).** The World Health Organization Quality of Life (WHOQOL) project was initiated in 1991, for the development of an international cross-culturally comparable quality of life assessment instrument. WHOQOL instruments have been developed collaboratively in a number of centres worldwide, and extensively field-tested. The WHOQOL-BREF instrument comprises 26 items, of which 2 concern overall perception of QoL and health, while the remaining 24 measures related to the following broad domains: physical health (7 items), psychological health (6 items), social relationships (3 items) and environment (8 items). The WHOQOL-BREF is a shorter version of the original tool and may be more convenient for use in large research studies or clinical trials. Item scores range from 1 to 5, with higher scores indicating better quality of life. Initial domain scores, obtained by multiplying the average score of domain items by 4, range from 4 to 20 and are then converted to a 0–100 scale. The WHOQOL-BREF takes under five minutes to complete, has been tested in several large samples [21–23] and is available in 19 languages. 11 studies using the WHOQOL-BREF questionnaire were included for analysis.

**The Duke Health Profile (DUKE) (n = 3)** was developed by Parkerson et al in 1990 [24]. The DUKE is a 17-item generic self-report instrument containing six health measures (physical, mental, social, general and perceived health and self-esteem), and four dysfunction measures (anxiety, depression, pain and disability). To obtain final scores of 0–100 per measure, the sum of the raw scores is divided by the maximum possible score and multiplied by 100, with high scores for health measures indicating good health and those of dysfunction measures indicating poor health. The DUKE has been translated into seventeen languages and as it requires five to 10 minutes to complete, it provides a rapid way of measuring health. Analysis by the present authors included 3 studies [25–27] and showed that the DUKE was principally chosen for its ease of use and variety of quality of life measures.

**15D (n = 3)**. In 3 studies analysed [28–30] quality of life was evaluated using the **15D**, a generic, 15-dimensional, standardized, self-administered HRQoL instrument that can be used both as a profile and a single index utility score measure [31] The questionnaire measures 5 levels of severity for each of the 15 dimensions: moving, seeing, hearing, breathing, sleeping, eating, speech, eliminating, usual activities, mental function, discomfort and symptoms, depression, distress, vitality and sexual activity. The single index score uses a 0–1 scale where 1 corresponds to no problems on any dimension. This questionnaire is well validated, has been shown to be reliable, sensitive and responsive to change [31,32] and takes an average of 5 to 10 minutes to complete [33]

**Ferrans & Powers Quality of Life Index (QLI) (n = 2).** In their study Rannestad et al. (2001) [34] use the QLI, first described in 1985. This index is composed of different items, scored on six-point Likert scales, relating to four specific life domains: °health/functioning, °psychological/spiritual, °socio/economic and family. The total score, ranging from 0–30, is calculated after adjustment of satisfaction responses according to response importance, with high scores denoting increased HRQoL. The QLI has been shown to have a high degree of validity and reliability [35] it is available in several languages and is widely used in clinical research.

#### Specific endometriosis scales

**EHP-30 (n = 43).** From 200 publications found to have used the EHP-30, 43 met inclusion criteria. The 30-item Endometriosis Health Profile (EHP-30) developed by Georgina Jones [4,7,36] is a specific HRQoL scale derived from interviews of patients with endometriosis. It consists of two parts: a 30-item core questionnaire which is applicable to all women with endometriosis, relating to five subscales (pain (11 items), control and powerlessness (6 items), emotions (6 items), social support (4 items) and self-image (3 items), and a second, 23-item modular questionnaire with six subscales, and some parts not applicable to all women such as for those who have no children (work life (5 items), relationship with children (2 items), sexual intercourse (5 items), the medical profession (4 items), treatment (3 items) and infertility(4 items)). Response categories are rated on a five-point Likert scale (0–4). Raw scores (the sum of items in each subscale) are translated into a score (each raw score is first divided by the maximum possible raw score and multiplied by 100) ranging from 0 (best possible health status) to 100 (worst possible health status). The EHP-30 is available in many languages [37–40]. If self administred, takes an average of 10 to 15 minutes to complete [37] and is the most extensively validated specific questionnaire for HRQoL measurement in women with endometriosis [36,41]. In EHP-30 validation studies, SF-36 is often used as the gold standard, and allows analysis of the convergent validity [38–40]. These studies report a significant correlation between the two scales.

**EHP-5 (n = 12)**. A total of 234 publications were found to have used EHP-5, of which 12 fulfilled inclusion criteria. The EHP-5 is a condensed version of the EHP-30 instrument, comprising one item with the highest correlation value, from each of the 5 EHP-30 scales. It consists of a 5-item core questionnaire about pain, control and powerlessness, emotions, social support, self-image and a 6-item modular questionnaire about work life, relation with children, sexual intercourse, medical profession, treatment and infertility. The response system consists of five levels ranged in order of severity: ‘never’, ‘rarely’, ‘sometimes’, ‘often’ and ‘always’. The short-form EHP-5 has been tested and shown to be highly correlated with the parent scale (EHP-30). It takes an average of 5 minutes to complete.

#### Self developed studies (specific scales)

**Colwell scale [42]**. One publication was found to use this scale and is described by the author as a 95-item HRQOL questionnaire with both generic and endometriosis-targeted scales and items. The scales include general health, comparative health, physical functioning, role functioning-physical, role functioning-emotional, bodily pain, anxiety, depression, behavioral/emotional control, general positive affect, and emotional ties/loneliness. Items and scales also included social functioning, endometriosis pain, overall health interference, health interference related to social and sexual functioning, symptoms and treatment satisfaction. A total of 137 women with endometriosis completed the questionnaire which was found to demonstrate good psychometric properties, with reliability, validity and responsiveness. No method relating to calculation of the HRQoL score or range for this scale was specified.

**Bodner scale [43]**. One publication was found using Bodner scale, described by the author as a 16-item HRQoL questionnaire. The scales included menstrual symptoms, side effects of medical treatment and impact of pelvic pain on functioning and well-being. 197 women with endometriosis completed the questionnaire which was found to demonstrate good psychometric properties, good reliability, validity and responsiveness in addition to a high correlation with SF-36 results. No method for calculation of the HRQoL score was specified.

**Medical Outcomes Study long form**: **Mathias et al** (1996) [44] reported using a self-developed questionnaire, incorporating a quality-of-life component based on the Medical Outcomes Study long form. The questionnaire was used to assess prevalence of chronic pelvic pain and its association with sociodemographic factors, quality of life, work productivity and use of health care resources. It included questions on pelvic pain severity, frequency and diagnosis (if any); health-related quality of life (general health, energy-vitality, pain interference, physical functioning, sexual functioning, emotional functioning, bed-days, reduced- activity days); indirect costs (employment status, wages, time lost from work, reduced productivity at work); and health care ressource use (visits to physicians or other health care providers, diagnostic or surgical procedures, use of medications) and demographics.

**Regidor et al. (**1997) [45] also report use of a four- page self-developed questionnaire for evaluation of pain symptom recurrence and infertility treatment in patients with endometriosis. It is available in English or German on request. Questions focus on occurrence of dysmenorrhea, dyspareunia, chronic pelvic pain or premenstrual pain occurring during the follow-up period, in addition to collecting data on the time of first appearance and intensity of pain symptoms (mild, moderate or severe) after therapy.. The second part of the questionnaire focuses on infertility outcomes, including duration of infertility, type of stimulation therapy after GnRH-agonist treatment, pregnancy rate, whether spontaneous or under stimulation programs, and rates for birth, miscarriage and ectopic pregnancies. The author can also gather information, via a semiquantitative scale, on patient subjective feelings relating to benefits and regain in quality of life post- therapy, as well as adverse side effects of GnRH-agonist therapy. The last part of the questionnaire deals with documenting medical or surgical therapies as a result of recurrence of endometriosis. Though this questionnaire allows evaluation of symptoms and HRQoL, the latter remains incomplete evaluation as only specifc areas are assessed.

**Oehmke et al (**2009) [46] report on use of a 51-question, self- developed questionnaire used to explore patient clinical histories, symptoms and impact of endometriosis on quality of life, physical and emotional well-being, social function, work-based and/or professional performance, participation in sexual intercourse and relation with partner. No details were provided on the method of calculation of the HRQoL score.

**Trehan et al**. (2009) [47] used a **self-developed questionnaire** created by incorporating the pain score, health status and quality of life 5-point visual analogue scale pictures from the Dartmouth Primary Care Cooperative Information Project Chart system quality of life questionnaire. It was developed using the framework of functional status measuring of the COOP charts, in order to be used easily in primary care practices, whatever the country is [48].

Kumar et al. (2011) [49] reported on use of the **Life Satisfaction Questionnaire,** developed by Carlsson and Hamrin (2002) [50]. Items are rated on a 6-point Likert scale where 1 corresponds to very dissatisfied and 6 to very satisfied. The mean of item scores is used to calculate a total life satisfaction score. The questionnaire takes on average 10–30 minutes to complete and has been used in chronic pain populations. For assessment of pelvic pain, the authors also used the Pelvic Pain Questionnaire, a blend of the Short-Form McGill Pain Questionnaire [51] and the Functional Pain Scale.

Learman et al. (2011) [52] reported use of a **HRQOL questionnaire** which allowed evaluation of the Pelvic Problem Impact Scale, Physical Component Summary score, Mental Component Summary score and beliefs about current health. The physical and mental component summary scales were based on the Medical Outcomes Study SF-12 questionnaire, details of which are provided in an earlier publication [53]

Ceccaroni et al. (2012) [54] used a 54-item **self-developed questionnaire to measure** quality of life, sexual functions (DSMIV criteria (Diagnostic and Statistical Manual of Mental Disorders IV)) and psychological status (based on the **Short WHOQoL** of OMS).

Issa et al. (2012] [55] reported use of a **Quality of Life Score,** previously described by Gonsalkorale et al in 2002 [56].

Fritzer et al. (2012) [57] used a **10-item questionnaire with a 4-point Likert scale,** based on standardised questionnaires such as the SF-36 Health Status for physical and mental health.

**Chapron et al. (**2015) [58] reported on the impact of endometriosis in women from three culturally and economically differing regions (China, France and Russia). The questionnaire used included three sections: 1) level of general awareness and knowledge of endometriosis, 2) methods of diagnosis for the various forms of endometriosis; and 3) impact of endometriosis on everyday activities. Depending on the answers to preliminary questions certain questions were asked only of specific sub-groups.

### Quality of scales: Qualitative analysis: Comparative analysis of scales

**Fig 2:**
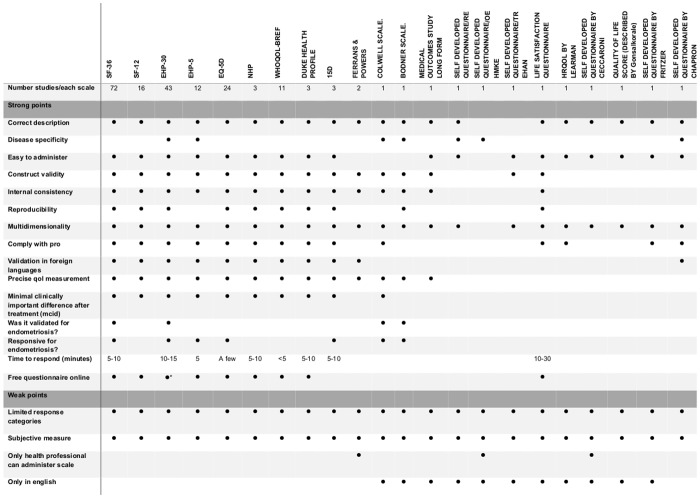
Characteristics of quality of life scales: Strengths and weaknesses.

Qualitative analysis was performed as previously described. The principal characteristics (classified as strong or weak of the Quality of Life scales) are summarized in Fig 2.

#### Scale description and characteristics

Most of the scales are accurately described in the literature with the exception of a few self- developed scales which tend to be used one-off in a single study. SF-36 was found to be used in 72 studies (Tables 1 and 2), and is the most frequently used HRQoL scale in endometriosis, followed by the EHP-30 scale, which was used in 41 studies. While detailed descriptions of how scales are implemented tend to be available in the literature, comparative analysis is made more difficult when authors administer scales such as NHP and SF-36 in varying formats [59] as in the case of Burry et al. (1992) [19] who adminstered a modified version of Part II of the NHP.

#### Validity, responsiveness, reproducibility and reliability

Validation of a questionnaire, requires comparison with other validated scales in a population, enabling confirmatory factor analysis in addition to analysis of internal consistency and reliability, construct and discriminant validity and responsiveness. HRQoL scales should be adequately validated, reproducible and reliable. The most commonly used scales are accurately validated (SF-36, SF-12, EHP-30, EHP-5, EQ5D, NHP, WHOQOL-BREF, DUKE HEALTH PROFILE, 15D) and reliable. Information on other psychometric properties, such as reproducibility (often obtained with the test-retest phase) or responsiveness (the ability to detect a response to treatment or a change in health status, which is of major importance) is in some cases provided (SF-36 [60], EHP-5 [61], [62] EHP-30 [4,38,63] E5-QD [61].

Only 10 scales have as yet not been validated or tested using reproducibility and reliability studies, most of which concern self-developed scales [43,45–47,52,54,55,57,58,64].

Some generic scales may be responsive to only certain diseases and not endometriosis such as the NHP. Only 3 scales were found to be responsive and able to detect a change in HRQoL score following endometriosis treatment (SF-36, EHP-30, EQ5D (Fig 2)). Information on construct validity and responsiveness may be incomplete as for the EHP-5 (not fully studied in the original version). However, the French version of the EHP-5 is reported as having good psychometric properties and to be an efficient, valid and responsive tool suitable for daily pratice [61,62].

For the EHP-30, the responsiveness was analysed by Jones [4] and by van de Burgt et al. [63]. The EHP-30 has been shown to be sensitive regarding measures of change in health status, with patients who reported improvement in health status showing statistically significant changes in scores.

EQ-5D is one of the HRQoL questionnaires most often used in endometriosis [61, 65] and it is recognised for its high sensitivity to change and has therefore been used in clinical studies that seek to evaluate the impact of surgery on quality of life in endometriosis patients [3,66].

#### Disease specificity, multidimensionality

Both generic and disease-specific instruments have been used to evaluate the impact of endometriosis on patient health-related quality of life.

However, generic questionnaires have been designed to measure health status across a wide variety of diseases and may be unable to collect information on areas of well-being and functioning that are important to women with endometriosis or be sensitive enough to assess changes. The SF-36, for example, is a tool frequently used to assess health-related quality of life in women with endometriosis, but fails to collect information on dyspareunia, a symptom that can give rise to considerable strain on personal relationships and impact emotional well-being. Disease-specific questionnaires that contain items developed with appropriate patient groups, are likely to be more responsive to changes in health status.

Those scales that address issues specific to endometriosis, issues which are overlooked by other scales, include EHP-30, EHP-5, Bodner scale and Colwell scale [42,43] and various self-developed questionnaires [45,46,58,64]. However the validity of these specific instruments may be questioned, especially where the majority of items [44,67] are not derived from patient feedback but taken instead from generic health-status questionnaires or clinicians. In contrast, other specific scales (such as the EHP-30 its shorter form the EHP-5, the Colwell scale, the Bodner scale, the scale developed by Thomassin, and the one by Regidor [42,43,45,64] have been designed on the basis of interviews of women with endometriosis [36] in accordance with health status questionnaires guidelines [10].

#### Respondent and investigator burden and feasibility

Most of the scales are easy to administer and demonstrate good patient compliance.

Some scales require adminstering by doctors or other health staff [46,67], whereas others can be self-administered so avoiding rater bias and reducing investigator time. Some such as the SF-36 or the EHP-30 use electronic self administration which facilitates both rapid completion and data collection. Furthemore more recent developments in technology enable questionnaire links to be shared widely via tools such as smartphone apps or social networks [38], also facilitating quick and large collection of data. Some scale are short (10–12 item to complete) and other are longer (>50 items) leading to different time to complete, and different use (research or daily clinical practise…).

Most of the scales require between 5 and 15 minutes to complete (Fig 2), with scales that use electronic means being the least time consuming.

#### Validation in foreign languages

A notable number of scales are available in languages other than English with the exception of the Colwell scale, the Bodner scale and a number of self-developed scales which only have an English-speaking version. This limits their use internationally as well as presenting obstacles for comparison between studies performed in different countries. All other scales are widely used, translated and validated in several languages. The most translated scales are the SF-36 and the EHP-30. The EHP-30 is the specific scale for endometriosis that has been translate in most languages [38,39,68,69]. The EHP-5 underwent cross-cultural adaption in three countries: France, Turkey and Iran [62,70,71].

#### Responder concept and Minimal Clinically Important Differences (MCID)

A patient is a responder when there is a score change in a measure, however small, experienced by this individual patient over a predetermined time period, that has been demonstrated in the target population to have a significant treatment benefit (U.S. Department of Health and Human Services FDA, 2009). In a study of the Dutch-version of the EHP-30, the MCID was reported to be equivalent to a change in score ranging between 3.2 and 17.5 units, depending on the dimension [63] Jones et al. [4] also evaluated minimally important differences and responsiveness of the EHP-30, before and after surgery for endometriosis.

Revicki et al. (2006) [72] caution that MCID may vary by population, context and type of statistical analysis, and that no one MCI would be valid for all study applications involving a PRO instrument. Responsiveness and MCID must be demonstrated and documented for the particular study population.

To date, little data is available in the literature regarding MCID for endometriosis and HRQoL scales. MCID has been described in endometriosis specifically for the pain scale [73], for the SF-36 [60], and the EHP-30 [63], for EHP-5 and EQ5D [61]. Stull et al. [60] report favorable results for the SF-36 measure demonstrating its responsiveness to change. On the basis of effect size the two dimensions of the SF-36 scale that demonstrate the capacity to detect treatment effects or differences are bodily pain (BP) and the physical composite score.

### Quality of Evidence assessment (Table 3)

**Table 3:** Quality of Evidence assessment, using in the Canadian Task Force.

Canadian Task Force N = 187	SF-36 n = 72	SF-12 n = 16	EHP-30 n = 43	EHP-5 n = 12	EQ-5D n = 24	NHP n = 3	WhoqolBref n = 11	Duke n = 3	15D n = 3
**I Evidence from randomized controlled trial(s)** **N = 45 = > 24,1%**	**20/72 = 27.8%** Agarwal et al., 2015 [100] Angioni et al., 2015 [98] Berner et al. 2015 [102] Caruso et al., 2015 [103] Caruso et al., 2016 [104] Darai et al, 2010 [111] Miller et al, 2000 [133] Rubi-Klein et al, 2010 [143] Gallagher et al, 2016 [144] Sesti et al, 2007 [145] Strowitzki et al, 2010 [148] Strowitzki et al, 2010 [149] Strowitzki et al, 2012 [150] Stull et al, 2014 [60] Tanmahasamut et al, 2012 [151] Teixeira et al, 2017 [152] Touboul et al, 2015 [77] Vercellini et al, 2002 [154] Zhao et al, 2012 [155] Zupi et al, 2004 [156]	**4/16 = 25%** Abbott et al, 2004 [66] Di Francesco et al, 2014 [160] Meissner et al, 2016 [165] Soto et al, 2017 [167]	**12/43 = 27.9%** Cheong et al, 2014 [202] Flower et al, 2011 [203] Jenkinson et al, 2008 [206] Wickström et al, 2013 [220] Ahn et al, 2009 [199] Al-Azemi et al, 2009 [200] Gallicchio et al, 2015 [204] Gonçalves et al, 2017 [205] Middleton et al, 2017 [210] Mira et al, 2015 [211] Soto et al, 2017 [167] Wayne et al, 2008 [219]	**2/12 = 16.7%** Acs et al, 2015 [221] Carr et al, 2014 [223]	**3/24 = 12.5%** Abbott et al, 2004 [66] Daniels et al, 2009 [171] The Luna trial collaboration, 2003 [183]	**2/3 = 66.7%** Bergqvist et al, 2001 [18] Burry et al, 1992 [19]	**1/11 = 9.1%** Zhao et al, 2013 [197]	**1/3 = 33.3%** Stratton et al, 2008 [167]	
**II-1 Evidence from controlled trial(s) without randomization** **N = 9 = > 4,8%**	**6/72 = 8.33%** Centini et al., 2013 [106] Chene et al, 2008 [107] Nnoaham et al, 2011 [85] Roman et al, 2012 [150] Siedentopf et al, 2008 [146] Ribeiro et al, 2014 [140]	**1/16 = 6.3%** Garry et al, 2000 [3]					**2/11 = 18.2%** Lee et al, 2016 [191] Lin et al, 2014 [193]		
**II-2 Evidence from cohort or case–control analytic studies, preferably from more than one centre or research group** **N = 90 = > 48,1%**	**30/72 = 41.7%** Angioni et al., 2015 [99] Bassi et al, 2011 [101] Bodner et al., 1997 [106] Chene et al, 2012 [108] Chuamoor et al, 2012 [109] Darai et al, 2015 [75] De Graaf et al., 2013 [112] De Graaf et al, 2015 [113] Di Donato et al, 2013 [115] Dubernard et al, 2006 [116] Hefler et al, 2005 [121] Jia et al, 2013 [40] Jones et al, 2004 [4] Jones et al, 2001 [36] Laursen et al, 2005 [124] Leplege et al, 1998 [14] Lövkvist et al, 2016 [125] Mabrouk et al, 2012 [126] Mabrouk et al, 2011 [127] Mabrouk et al, 2011 [128] Maiorana et al, 2012 [129] Piketty et al, 2007 [131] Melis et al, 2014 [132] Morotti et al, 2014 [135] Nogueira-Silva et al, 2015 [69] Nojomi et al, 2011 [39] Nunes et al, 2014 [136] Remorgida et al, 2007 [139] Silveira da Cunha Araùjo et al, 2014 [147] Tu et al, 2014 [153]	**8/16 = 50%** Abbott et al, 2003 [157] Carey et al, 2014 [158] Lyons et al, 2006 [164] Fourquet et al, 2011 [162] Moawad et al, 2011 [166] Facchin et al, 2015 [161] Luisi et al, 2015 [163] Zupi et al, 2015 [169]	**23/43 = 53.5%** Hansen et al, 2014 [86] Jones et al, 2001 [36] Jones et al, 2004 [4] Jones et al, 2004 [207] Jones et al, 2006 [7] Vercellini et al, 2013 [218] Van de Burgt et al, 2011 [41] Van de Burgt et al, 2013 [63] Tan et al, 2013 [216] Nojomi et al, 2011 [39] Meuleman et al, 2014 [209] Chauvet et al, 2017 [38] Hansen et al, 2013 [85] Jia et al, 2013 [40] Kent et al, 2016 [185] Khong et al, 2010 [82] Maiorana et al, 2012 [129] Meuleman et al, 2011 [208] Meuleman et al, 2009 [5] Nogueira-Silva et al, 2015 [69] Protopatas et al, 2014 [212] Shakir et al, 2015 [188] Van der Houwen et al, 2014 [217]	**8/12 = 66.7%** Bailly et al, 2013 [170] Fourquet et al, 2011 [162] Goshtasebi et al, 2011 [70] Jones et al, 2004 [207] Minas et al, 2014 [225] Fauconnier et al, 2017 [62] Renouvel et al, 2009 [226] Selcuk et al, 2015 [71]	**14/24 = 58.3%** Kent et al, 2016 [185] Shakir et al, 2015 [188] Ford et al, 2004 [174] Garry et al, 2000 [3] Lyons et al, 2006 [164] Radosa et al, 2014 [179] English et al, 2014 [173] English et al, 2007 [172] Klein et al, 2014 [176] Michalak et al, 2016 [186] Radosa et al, 2016 [187] Roman et al, 2010 [180] Roman et al, 2010 [181] Simoens et al, 2011 [182]	**1/3 = 33.3%** Clarke et al, 1995 [20]	**5/11 = 45.4%** Cao et al, 2015 [189] Giuliani et al, 2016 [91] Leonardo-Pinto et al, 2017 [192] Tripoli et al, 2011 [92] Yela et al, 2015 [196]	**1/3 = 33.3%** Stratton et al, 2015 [26]	
**II-3 Evidence from comparisons between times or places with or without the intervention; dramatic results from uncontrolled studies could be included here** **N = 37 = > 19,8%**	**15/72 = 20.8%** Augusto et al., 2016 [100] Caruso et al., 2015 [105] Deguara et al, 2013 [114] Dubernard et al, 2008 [6] Farfaras et al, 2014 [117] Friedl et al, 2015 [118] Friggi Sebe Petrelluzzi et al., 2012 [119] Grandi et al, 2015 [120] Hong et al, 2014 [122] Petrelluzzi et al, 2008 [137] Laas et al, 2015 [123] Marques et al, 2004 [130] Montanari et al, 2013 [134] Pontis et al, 2016 [138] Roman et al, 2015 [141]	**3/16 = 18.8%** Deguara et al, 2013 [114] De Graaf et al, 2016 [168] Vercellini et al, 2016 [168]	**5/43 = 11.6%** Benbara et, 2008 [201] Friedl et al, 2015 [118] Meuleman et al, 2011 [178] Selvi-Dogan et al, 2016 [214] Soliman et al, 2017 [215]	**1/12 = 8.3%** Boileau et al, 2012 [222]	**6/24 = 25%** Bailly et al, 2013 [170] Bluett et al, 2016 [184] Fritzer et al, 2014 [175] Li et al, 2014 [177] Meuleman et al, 2011 [178] Simoens et al, 2012 [8]		**3/11 = 27.3%** Kiykac Altinbas et al, 2015 [190] Sepulcri et al, 2009 [194] Souza et al, 2011 [195]	**1/3 = 33.3%** Karp et al, 2011 [25]	**3/3 = 100%** Taipale et al, 2009 [30] SetäLä et al, 2012 [28] Kössi et al, 2013 [29]
**III Opinions of respected authorities, based on clinical experience; descriptive studies or reports of expert committees** **N = 6 = > 3,2%**	**1/72 = 1.4%** Darai et al, 2009 [110]		**3/43 = 6.98%** Darai et al, 2009 [115] Gao et al, 2006 [81] Rostami et al, 2015 [213]	**1/12 = 8.3%** Goyal et al, 2016 [224 ]	**1/24 = 4.2%** Gao et al, 2006 [81),				

Most of the studies included were classified II-2 (i.e. Evidence from cohort or case–control analytic studies, preferably from more than one centre or research group, N = 90, 48,1%).

If we look at the most frequently used scale, for the SF-36 most of the studies included were also classified II-2 (n = 30, 41.7%), and for the EHP-30 more than the half were classified II-2 (n = 23, 53.5%).

## Discussion

This review shows that the two scales most frequently used are the SF-36 and the EHP-30, and that the most validated scales were SF-36 and EQ-5D for generic questionnaires and EHP30 and its short form the EHP-5 for the specific ones. Both generic and disease-specific instruments have been used to evaluate the impact of endometriosis on patient health-related quality of life. However, as generic questionnaires were designed to measure health status across a wide variety of diseases, the psychometric properties and internal consistency levels are not sufficiently established for the measurement of HRQoL with endometriosis [59]. In addition these questionnaires do not collect information on all areas of well-being and functioning important to women with endometriosis, so reducing their potential to assess changes. One example of this is the failure of SF-36 to evaluate dyspareunia, which is known to have a frequent and negative impact on emotional well-being and personal relationships of women [74–76]. Disease-specific instruments are more sensitive to disease experiences than generic instruments [77]. They contain items developed from appropriate patient groups and are more responsive to changes in health status. Generic scales however allow comparisons across diseases, and between patient scores and those of the general public. In this way HRQOL of women with endometriosis can be compared with HRQOL experiences linked to other diseases.

The present authors found that the questionnaires adminstered most often in endometriosis are SF-36 and EHP-30, in accordance with the litterature [78]. Jones et al (2004) [4] report how these scales measure similar attributes, highlighting similitudes between the SF-36 bodily pain scale and the EHP-30 pain dimension, and between the SF-36 mental health scale and the EHP-30 emotional wellbeing dimension. However, caution is required when comparing the SF-36 social functioning dimension and the EHP-30 social support scale which despite their similar sounding terms, measure different attributes. Indeed where the social functioning scale measures the impact of illness on patient ability to continue with social activities, the EHP-30 scale measures the impact of endometriosis upon a woman’s social support network. Despite these differences in most EHP-30 validation studies, the SF-36 is frequently used as the gold standard [38–40] and analysis reaveals significant correlation between the two scales. SF-36 has been specifically validated for endometriosis [60] while EHP-30 is specific to endometriosis [36] and is recommended by several medical societies [59,79].

Apart from the well-known EHP-30 and EHP-5 scales, 6 other disease-specific questionnaires have been developed for endometriosis [42,43,45,46,58,64]. Caution is required when considering the validity of these instruments in particular due to the way items are taken from generic health-status questionnaires or created by clinicians and not derived from patients with the condition. In contrast the EHP-30 has been designed using data from interviews of women with endometriosis [36] and as a consequence the questionnaire dimensions better reflect the many aspects of well-being and functioning affected by the disease.

### The SF-36 a gold standard?

In validation studies, the SF-36 is often used as the gold standard and allows analysis of convergent validity [38–40] Significant correlations were found between EHP-30 scales and similar SF-36 scales as hypothesised and good expected negative associations were found, in line with findings byJones et al. (2001) [36]. Dubernard et al. (2008) [80] (n = 36) put forward the SF-36 questionnaire as a tool capable of predicting the degree of change in HRQoL after laparoscopic management of posterior DIE (deep infiltrating endometriosis), delineating a new approach of DIE in which HRQoL evaluation might guide the management of the disease. Dubernard et al. considered an improvement to occur when the postoperative score improved by 1 SD (based on the preoperative score of the training set) when compared with the preoperative score. The results confirmed that preoperative SF-36 scores can be used to predict the degree of change in HRQoL after laparoscopic segmental colorectal resection for endometriosis. Women with preoperative Physical Component Summary (PCS) and Mental Component Summary (MCS) scores below 37.5 and 44.5, respectively, had 80.7% and 84.2% probabilities of seeing their scores improve after surgery, whereas women with preoperative scores above 46.5 and 47.5, respectively, had probabilities of 0% and 10.7% for improving scores. Valentin et al. (2017) [81] also used SF-36 preoperative scores for predicting improvement in quality of life following laparoscopic management of minimal endometriosis (n = 167), defining improvement in PCS or MCS subscales as an increase of 5 points. The results of this study led to the establishing of two thresholds for the SF-36 i.e. 50 for PCS and 40 for MCS, above which risk of failure is very high (86% failure in our population), and below which risk of failure remains high (54.3%).

In conclusion the SF-36 additionally provides a simple tool for practitioners wishing to select and inform women who might benefit from laparoscopic treatment of endometriosis.

### Which scale to use?

The EHP-30 seems to be the most reliable and most thoroughly validated questionnaire for HRQoL measurement in women with endometriosis [36,41] It exhibits good reliability, validity and interpretability [4,7,36] and has been recommended in HRQoL research on endometriosis by both the American Society for Reproductive Medicine and the European Society for Human Reproduction and Embryology [59].

Weaknesses of the EHP-30 include the length of the questionnaire. The time required to fill out the questionnaire is evaluated at between 10 and 15 minutes and although the EHP-30 may provide a thorough HRQoL evaluation, the completion time needs to be shortenened, so that it can be completed outside of consultations. Furthermore, the EHP-30 is not appropriate for use with the general population, although it has been administered in two studies [82,83] with some adaptations. Most of all the EHP30 does not allow to calculate a single aggregated index but instead 11 dimensions that can vary differently [63]. It may thus complicated to use this score as a single outcome in clinical study. The EHP-5, on the other hand derived from the EHP-30, is shorter and thus could fulfill a role in evaluation of HRQoL in everyday clinical practice. It has been demonstrated that the 5+6 questions of the EHP-5 were completely one-dimensional with very high internal consistency [62]. It allows providing a single overall QoL score to measure how endometriosis impairs daily life.

In comparison the SF-36 and EQ-5D are not a specific questionnaires, but as they allow comparisons across diseases and between patient scores and those of the general public. EQ-5D, as a generic instrument, is particularly useful for epidemiological studies since it allows comparison of managements or populations regardless of the disease [84]. EQ5D might also allow economic evaluation of health-care interventions [8]. The choice of a particular scale will depend to some extent on the weight of available evidence concerning its capacity for measurement in the specific context of women affected by endometriosis.

### MCID

Vincent et al. (2010) [59] suggested that the definition of a responder in endometriosis corresponds to a >30% or >50% reduction in symptoms, the precise definition being trial dependant, and therefore this should be clearly defined for each trial.

The concept of MCID allows a more precise and probably more valuable method for distinguishing responders from non-responders. MCID after treatment is considered to be ‘the smallest difference in score in the domain of interest that patients perceive as important, either beneficial or harmful, and that would lead the clinician to consider a change in the patient’s management’ [72,73]. Furthermore, there are many different analysis to calculate and evaluate MCID [74].

Treatment options are limited in endometriosis and it is important therefore to evaluate, from the patient’s perspective, the ways in which different treatments affect HRQoL. Analysis of MCID is available in the literature for medical treatment but not as yet for surgical treatment. MCID has been described in endometriosis specifically for the pain scale [76], but to date, little data is available in the literature regarding MCID for endometriosis and HRQoL scales ([60] [63] [61]). However endometriosis is a complex disease and although pain is an important component of the syndrome it is not the only one [28]. In this context, scales that only assess pain intensity in endometriosis remain incomplete. A fuller picture of endometriosis as a disease requires consideration of HRQoL, and notably that from the patient’s point of view [85].

To date no single method for establishing MCID is considered ideal or accepted and many varying assumptions are made concerning change.

Health status constructs are generally based on multi-item scales. These scales are derived from individual item responses to a questionnaire that are then summed with or without weighting. Both multi- and single-item health status scales are expressed in units of measurement that have no direct biological meaning [86]. Clinically important change is a relevant concept for the provider as well as the patient. An important change for the patient may be one that represents a meaningful reduction in symptoms or improvement in function.

The main problem concerns the concept of Responder and MCID. The value of MCID is specific to the population, to the lapse of time between the two evaluations and is disease specific.

In a study on SF-36 validation in patients with endometriosis, Stull et al. (2014) [60] focused on minimally important difference and effect size. Two SF-36 dimensions, the BP subscale and PCS, performed well in detecting treatment effects and differences. The responsiveness of the SF-36 to detecting improvements, suggest that reported changes in SF-36 in the context of a clinical trial based on target MCID, such as those previously mentioned, are likely to be meaningful. A similar conclusion could be made for the EHP-30 based on Van de Burgt et al’s (2013) evaluation of the MICD with the Dutch version. Except for the EQ-5D et EHP-5, for the other HRQoL scales, target MCID are yet to be established for endometriosis. According to Yost, no single method of establishing an MCID is ideal or accepted and each one makes certain assumptions about change [87]. Consequently, researchers should use multiple methods and triangulation of consistent values or those within a consistent range across methods.

### Other specific scales

Some studies in our review used other specific scales for assessment, in addition to a HRQoL scale. Firstly in the case of sexual functionning, studies used the *McCoy Female Sexuality Questionnaire (MFSQ)*, a scale that evaluates sexual experiences in the previous four weeks by way of seven-point Likert scales for various aspects of sexual life [28,29,88] or the Golombok Rust Inventory of Sexual Satisfaction (GRISS) [89]. Secondly in other studies, scales such as the Hospital Anxiety Depression Scale (HAD) were used for assessing mental health-related diseases. Finally digestive symptoms, frequently associated with endometriosis, were assessed using the ROME III diagnostic questionnaire, the Irritable Bowel Syndrome Symptom Severity Score (IBS SSS), or the Non-Colonic Symptom and Quality of Life Score [55].

Certain studies chose to focus on a specific dimension, such as the impact of the disease on work, rather than on global HRQoL. Thus Wullschleger et al. [90] reported on the impact of minimally invasive surgery for endometriosis on health and on quality of work life. Nnoaham et al. (2011) [85] focused on the impact of endometriosis on quality of life and work productivity, and Hansen et al. (2013) [82] considered the influence of endometriosis-related symptoms on work life and work ability.

Similarly, Santuli et al. chose to focus on the dimension ‘fertility’ and used the Fertility Quality of Life tool to assess the impact of assisted reproduction techniques on painful symptoms and quality of life [91].

Two studies preferred a much simplified method for evaluation of HRQoL. Barrueto et al. (2015) [92] used reponses to a single question: “In general, would you say your health is excellent, very good, good, fair, or poor?”, while Roos-Eysbouts et al. (2015) [93] applied a scale of 1–10 to assess patient social life, health and quality of life. Both methods appear inadequate in view of current recommendations that highlight the importance of systematic, complete and appropriate evaluation of quality of life.

### Quality of life in the general population

Various norm-based scores are available for comparing quality of life in women with endometriosis with a general population; notably official norm-based scores derived from the 1998 US general population by Qualitymetric Incorporated [94] or those in languages other than English such as that of Leplege, France [14]. The aforementioned scores, collected more than 15 years ago, only included women from one country and as to date no updated or more recent norm-based scores have been made available, it is currently difficult to make well-founded comparisons with the general population.

### Weakness of our study

Included in this review were clinical trials, comparative studies, controlled clinical trials, RCTs and multicenter studies and excluded were all other published articles on treatment of endometriosis and HRQoL, including abstracts and congress presentations. As such relevant scales published in other articles may have been left out from the analysis. The methodology employed in this review is similar to that used by Bourdel et al [1] in a study investigating endometrisis and pain scales. The present authors limited research of articles to the field of endometriosis and HRQoL.

### Proposal for an ‘optimal’ HRQoL scale in endometriosis?

Analysis of the literature raises the question as to whether we need an ideal HRQoL scale. An optimal HRQoL scale in endometriosis would be required to take into account the specificities of endometriosis symptoms such as menstrual pattern and dyspareunia. It should be validated, reliable and adequately described, uniformly administered, easy and quick to administer and score and allow self-administration accessible to low literacy patients. Furthermore an optimal scale should provide for the concept of responder feasible and MCID, whilst allowing detection of comorbidities, such as dyschezia. Finally it should also allow daily assessment of HRQoL and be available in many languages.

SF-36 and EHP-30 scales appear well-balanced in terms of strengths and weaknesses (Fig 2), being validated, reliable, precise, sensitive to change, available in the most frequently spoken languages and easy to administer and complete. EQ-5D and EHP-5 have also important stenght points, and the great quality to be short and really easy to use in daily clinical practice.

Nonetheless the shorter response time associated with the SF-36 makes it easier to complete and administer in comparison with the EHP-30, in addition comparisons across diseases and between patients’ scores with those of the general public are possible, allowing cost-impact evaluation. Conversely, EHP-30 is specific, is likely to be more sensitive to disease experiences [77] and provides a more complete HRQoL evaluation. We believe this scale to be the best adapted for investigation of endometriosis. The main weakness of the EHP-30 concerns response time, making it difficult to complete during consultation time. In comparison the EHP-5 is shorter, time-saving and may be easier to use in everyday clinical practic. The EHP-30 provides a more complete and rigourous evaluation of HRQoL which is essential in clinical research, and appears to fulfill requirements for state-of-the-art PRO tools, its items being developed on the basis of patient input. A further important factor however, concerns the potential recall bias (4-week period) for the EHP-30 scale [95]. Bearing this in mind, the shorter EHP-5 remains useful for specific evaluation in everyday practice.

In the authors’ opinion it is not indispensable to use the EHP-30 in combination with the SF-36, as the latter does not allow assessment of the most important dimensions for endometriotic patients which are assessed by the EHP-30. Futhermore, significant correlations were found between the two scales for common themes.

## Conclusion

Generic scales allow both comparisons across diseases and between patient scores with those of the general public. In addition they allow comparisons of HRQOL in women with endometriosis with HRQOL linked to other diseases.

In clinical practice, routine evaluation of HRQOL in women who suffer from endometriosis is essential both for the health-care provider and the patient [96]. Studies should complete their analyses by providing information on MCID for the population studied; the proportion of patients reaching mininum MCID, items showing the most important improvements and those demonstrating no change. Ideally studies should measure HRQoL at least one calendar month before treatment and at 3, 6 and 12 months thereafter, continuing evaluation on an annual basis for as long a period as possible. The SF-36 scale is the most commonly used scale in endometriosis studies. Investigation of inherent strengths and weaknesses reveal good overall performance by both the SF-36 and EHP-30 when compared with other scales.

## Supporting information

S1 Checklist Prisma checklist. (DOCX)
